# Changes in the Utilization of Outpatient and Visiting Dental Care and Per-attendance Care Cost by Age Groups During COVID-19 Pandemic Waves in Japan: A Time-series Analysis From the LIFE Study

**DOI:** 10.2188/jea.JE20230323

**Published:** 2024-11-05

**Authors:** Taro Kusama, Yudai Tamada, Megumi Maeda, Fumiko Murata, Ken Osaka, Haruhisa Fukuda, Kenji Takeuchi

**Affiliations:** 1 Division of Statistics and Data Science, Liaison Center for Innovative Dentistry, Tohoku University Graduate School of Dentistry, Miyagi, Japan; 2 Department of International and Community Oral Health, Tohoku University Graduate School of Dentistry, Miyagi, Japan; 3 Department of Preventive Medicine, Nagoya University Graduate School of Medicine, Aichi, Japan; 4 Department of Health Care Administration and Management, Kyushu University Graduate School of Medical Sciences, Fukuoka, Japan

**Keywords:** infectious disease, health service, geriatrics, oral health

## Abstract

**Background:**

The influence of the coronavirus disease 2019 (COVID-19) pandemic on dental care utilization may have differed according to individual characteristics or type of dental care provision. This study aimed to evaluate the changes in dental care utilization and per-attendance costs by age group and type of dental care during the COVID-19 pandemic in Japan.

**Methods:**

This time-series study used healthcare insurance claims data from 01/07/2019 to 09/27/2021 (143 weeks) from nine municipalities in Japan. Dental care utilization rate per week and average dental care cost per attendance by age groups (0–19 years/20–64 years/65–74 years/≥75 years) and types of dental care (outpatient/visiting) were used as outcome variables. COVID-19 pandemic waves in Japan were used as predictors: 1^st^ (03/23/2020–05/17/2020), 2^nd^ (06/22/2020–09/27/2020), 3^rd^ (10/26/2020–02/21/2021), 4^th^ (02/22/2021–06/07/2021), and 5^th^ (07/05/2021–09/13/2021) waves. Fixed-effects models were employed to estimate the proportional changes.

**Results:**

In the fixed-effects model, we observed large declines in dental care utilization during the 1^st^ (17.0–22.0%) and 2^nd^ waves (3.0–13.0%) compared to the non-pandemic wave period in all age groups. In contrast, the average dental care cost per attendance increased in all age groups by 5.2–8.6% during the 1^st^ wave.

**Conclusion:**

During the initial wave of the COVID-19 pandemic in Japan, dental care utilization decreased in all age groups, whereas the average dental care cost per attendance increased. The COVID-19 pandemic may have changed the dental care provision pattern towards less frequent and more concentrated dental care to avoid the risk of infection.

## INTRODUCTION

The coronavirus disease 2019 (COVID-19) pandemic caused by severe acute respiratory coronavirus 2 (SARS-CoV-2) not only affected the health of the global population,^1^ but also influenced the utilization of health care services due to its high transmissibility.^2^ During the COVID-19 pandemic, health care service utilization decreased by 37% worldwide compared to the pre-pandemic periods.^2^ The risks of COVID-19 severity and mortality are known to be higher among older adults and those with comorbidities.^3^ Previous studies have reported that physician visits were avoided by high-risk patients during the COVID-19 pandemic.^4^

In dental settings, initial reports suggested a risk of COVID-19 transmission during dental treatment because dental treatment often includes aerosol-generating procedures.^5^ Previous studies reported dental visit avoidance during the COVID-19 pandemic periods.^6^^–^^9^ Research conducted in Japan reported that the decrease in dental visits was limited to the short term after the outbreak.^10^ Although the avoidance of dental visits is considered to differ according to the patient’s susceptibility to SARS-CoV-2 infection (eg, age or comorbidity), few previous studies have investigated the heterogeneity of dental visit avoidance during the COVID-19 pandemic by the patients’ characteristics. In addition, most previous studies have mainly evaluated outpatient dental care, and few have considered visiting dental care. The majority of patients who utilize visiting dental care are older adults who have limited activities of daily living and are comorbid with severe diseases or disorders. Although these patients are at high risk of COVID-19, routine oral care is essential to prevent aspiration pneumonia.^11^

We hypothesized that the magnitude of the change in dental care utilization differed by age group, especially for older adults, during the COVID-19 pandemic, and that the decline was evident in visiting dental care compared to outpatient dental care. The present study aimed to evaluate the change in outpatient and visiting dental care utilization and its cost per attendance during the COVID-19 pandemic in different age groups using healthcare insurance data in Japan.

## METHODS

### Study design and participants

This time-series study was based on healthcare insurance claims data from the National Health Insurance and Latter-Stage Elderly Healthcare System in Japan. The National Health Insurance includes those who were not covered by employee insurance (ie, self-employed, unemployed, or employees of smaller companies) and aged <75 years. Almost a quarter of the Japanese population is included in the National Health Insurance, and the proportion increases by approximately 70% for individuals between 65 and 74 years of age. The Latter-Stage Elderly Healthcare System includes all citizens aged ≥75 years except for those receiving welfare.^12^ Therefore, our targeted population in this study was relatively older than the general Japanese population. We obtained healthcare insurance claims data from nine municipalities across Japan. These data were obtained from the Longevity Improvement & Fair Evidence (LIFE) Study, the details of which have been described previously.^13^ The LIFE Study is conducted anonymously; therefore, it is not possible to publish the name of the municipality or information from which the name of the municipality can be deduced. Among all municipalities' data included in the LIFE study, we used the data from nine municipalities because the data of several municipalities could not be used for the present study aim and did not cover study periods. The total data collection period in the present study was 143 weeks (01/07/2019–09/27/2021). Original data were obtained as daily data for each insured person and were converted to weekly data to eliminate fluctuations in dental utilization by day of the week. We defined Monday as the start of the week, based on ISO-8601.

### Outcome variables

The number of weekly dental care utilizations was used as the outcome variable. In Japan, visiting dental care is provided by the health insurance system as well as outpatient dental care.^14^ We evaluated the utilization of outpatient and visiting dental care separately for those aged ≥65 years. For those aged <65 years, we summed both outpatient and visiting dental care utilization because the number of those who utilized visiting dental care was small (<1.0%). The number of weekly dental care utilizations was identified based on the dental practice codes presented in eTable 1. The number of weekly utilizations of dental care was counted by the age groups (0–19 years, 20–64 years, 65–74 years, and ≥75 years). The dental care utilization rate was calculated by dividing the number of weekly utilizations of dental care by the insured population of each municipality by age groups. The age of each participant was obtained monthly. The outcome variables of dental care utilization in the present study were total dental care in 0–19 years and 20–64 years, outpatient dental care in 65–74 years, visiting dental care in 65–74 years, outpatient dental care in ≥75 years, and visiting dental care in ≥75 years.

We also used the average dental cost per dental attendance per week, which was calculated by summing each patient's medical fee points within a dental attendance and averaging them within a week. The average dental cost per dental attendance was also created across age groups (0–19 years, 20–64 years, 65–74 years, and ≥75 years). For one municipality, healthcare insurance claims data for 0–19 years were not available; therefore, the outcome variable among the 0–19-years age groups was obtained from eight municipalities.

### Predictors

The COVID-19 pandemic waves in Japan were used as primary predictors. Although the first case of COVID-19 in Japan was confirmed on 1/16/2020, we focused on pandemic waves with a substantial number of confirmed cases. We defined waves of the COVID-19 pandemic (1^st^–5^th^ wave) based on the daily number of newly confirmed COVID-19 cases from the Japanese Government’s statistics (https://www.mhlw.go.jp/stf/covid-19/open-data.html). The periods of each wave are defined as follows: 1^st^ (03/23/2020 to 05/17/2020/), 2^nd^ (06/22/2020 to 09/27/2020), 3^rd^ (10/26/2020 to 02/21/2021), 4^th^ (02/22/2021 to 06/07/2021), and 5^th^ (2021/07/05 to 2021/09/13) waves. The reference period was the non-pandemic wave period, which excluded the periods of 1^st^–5^th^ waves from the total study period (01/07/2019–09/27/2021). The trends in the daily number of newly confirmed COVID-19 cases in Japan during each pandemic wave period are presented in eFigure 1. The government declared three states of emergency in Japan to discourage unnecessarily leaving home three times, although they were not legally binding.^10^ Each state of emergency was included in 1^st^, 3^rd^, and 4^th^ wave of the COVID-19 pandemic in Japan.

To fit temporal trends, we included continuous weeks from the start of the data period as linear trends. In addition, to consider seasonality, we also included two sine and cosine waves of one-year and half-year periods: sin(2π^*^t/(365.25/7)), cos(2π^*^t/(365.25/7)), sin(2π^*^t/((365.25/2)/7)), and cos(2π^*^t/((365.25/2)/7)), respectively. These temporal and seasonal trends were also included in the time-series model used in a previous study.^15^ We also considered the number of holidays each week by including the categorical variables of days of holiday in each week, because dental clinics usually do not accept patients on holidays, except for emergency cases in Japan. We present the simplified equation of the fixed-effects model used in the present study (1).

$$\begin{array}{rl} Y_{it}-{\bar{Y}}_{i} & =\beta_{1}({Wave}_{it}-{\bar{Wave}}_{i})\\ & \;+\beta_{2}({TemporalTrend}_{it}-{\bar{TemporalTrend}}_{i})\\ & \;+\beta_{3}({SeasonalTrend}_{it}-{\bar{SeasonalTrend}}_{i})\\ & \;+\beta_{4}({Holidays}_{it}-{\bar{Holidays}}_{i})+(u_{it}-{\bar{u}}_{i}) \end{array}$$ (1)

### Statistical analysis

We employed a fixed-effects model to assess the association between COVID-19 pandemic waves and changes in dental care utilization. The fixed-effects model can cancel out the influence of time-invariant covariates within each municipality, contributing to estimating less confounding associations.^16^ We fitted the negative binomial regression model with the outer product of the gradient vectors for standard errors and used unconditional maximum likelihood estimation, including dummy variables of each municipality.^17^ The model also included offset terms of the insured population, and rate ratios and 95% confidence intervals were estimated. The number of insured individuals by age group in each municipality is reported annually in the governmental statistics. The government statistics report the number of insured populations of the National Health Insurance and Latter-Stage Elderly Healthcare System at the end of September. Therefore, we linearly imputed the interregnum between the statistical time points. The number of insured populations in each municipality by age group is presented in eFigure 2, eFigure 3, eFigure 4, and eFigure 5. For the average dental cost per dental attendance, we fitted the linear regression model with sandwich estimators for standard errors and conducted a fixed-effects model with conditional maximum likelihood estimation, considering municipalities’ fixed effects. We estimated the differences in average dental costs per dental attendance and 95% confidence intervals. In addition, we used a log-transformed average dental cost per dental attendance to evaluate the relative changes in each period. We found that dental care utilization data were missing during several periods in some municipalities (eFigure 6). These missing data were considered to be generated due to errors in the data extraction procedure, and the missing mechanism was considered to be completely random; therefore, we conducted multiple imputations to address missing data.^18^ Missing values were imputed using multivariate imputation by chained equation, and 50 imputed data sets were created. The estimates obtained from each imputed data set were combined based on Rubin’s rule. For sensitivity analysis, we also conducted a complete records analysis. Statistical significance was set at alpha = 0.05. Stata/MP (version 17.0; Stata Corp., College Station, TX, USA) was used to perform the statistical analyses.

### Ethical issues

This study was approved by the Kyushu University Institutional Review Board for Clinical Research (approval number: 22114-02) and the Ethics Committee of Tohoku University Graduate School of Dentistry (approval number: 23835). Approval for data usage was obtained from the Personal Information Protection Review Board of each municipality.

## RESULTS

Descriptive statistics of average dental care utilization per week and dental care cost per attendance among the nine municipalities are presented in Table 1 and Table 2, respectively. The average dental care utilization per week decreased during the 1^st^ wave of the COVID-19 pandemic and recovered gradually in subsequent waves. eFigure 7, eFigure 8, eFigure 9, eFigure 10, eFigure 11, and eFigure 12 show a temporal change in the average dental care utilization by municipality. At the municipality level, we also observed a large decline in dental care utilization during the 1^st^ wave of the COVID-19 pandemic, irrespective of age group or type of dental care utilization. The decline in dental care utilization recovered in subsequent pandemic waves. Regarding the average dental care cost per utilization, we did not observe obvious changes during the various pandemic waves. eFigure 13, eFigure 14, eFigure 15, and eFigure 16 show a temporal change in the average dental care cost per utilization by municipality. The dental care cost peaked during the New Year’s holiday, and other fluctuations during these periods were unclear from the shape of the graph.

**Table 1.** Overall dental care utilization rate among all nine municipalities during each COVID-19 pandemic wave by age group and type of dental care utilization

Dental care utilization rate per 100 person-weeks		Non-pandemic wave period		1^st^ wave		2^nd^ wave		3^rd^ wave		4^th^ wave		5^th^ wave	
		Mean	SD	Mean	SD	Mean	SD	Mean	SD	Mean	SD	Mean	SD
Age group	Types of dental utilization												
0–19 years^a^	Total	3.97	1.15	3.06	1.25	3.58	0.92	4.13	1.11	3.98	0.95	4.41	1.13
20–64 years	Total	5.48	1.21	4.13	1.13	5.17	1.15	5.34	1.22	5.33	0.91	5.39	1.04
65–74 years	Outpatient	9.41	2.01	6.88	2.01	8.22	1.77	8.61	2.05	8.51	1.35	8.28	1.71
	Visiting dental care	0.26	0.07	0.18	0.07	0.24	0.07	0.25	0.09	0.23	0.07	0.27	0.07
≥75 years	Outpatient	7.89	1.73	5.93	1.68	6.50	1.41	7.26	1.60	7.60	1.22	6.84	1.32
	Visiting dental care	1.91	0.61	1.36	0.60	1.61	0.64	1.76	0.66	1.65	0.54	1.77	0.58

COVID-19, coronavirus disease 2019; SD, standard deviation.

^a^For the age group of 0–19 years, data were collected from eight municipalities.

Periods of pandemic waves were defined as follows: 1^st^ wave (03/23/2020–05/17/2020), 2^nd^ wave (06/22/2020–09/27/2020), 3^rd^ wave (10/26/2020–02/21/2021), 4^th^ wave (02/22/2021–06/07/2021), and 5^th^ wave (07/05/2021–09/13/2021).

**Table 2.** Overall average dental care cost per attendance among all nine municipalities during each COVID-19 pandemic wave by age group

Average dental care cost per attendance (JPY)	Non-pandemic wave period		1^st^ wave		2^nd^ wave		3^rd^ wave		4^th^ wave		5^th^ wave	
	Mean	SD	Mean	SD	Mean	SD	Mean	SD	Mean	SD	Mean	SD
Age group												
0–19 years^a^	6,792	1,782	6,401	734	6,470	605	7,806	2,303	6,748	618	6,739	635
20–64 years	6,959	1,783	6,713	429	6,774	449	8,142	2,463	6,867	457	6,923	466
65–74 years	6,655	1,662	6,489	508	6,658	492	7,855	2,225	6,720	493	6,757	492
≥75 years	6,918	1,507	6,782	470	6,909	494	7,913	2,087	7,015	432	7,070	464

COVID-19, coronavirus disease 2019; JPY, Japanese yen; SD, standard deviation.

^a^For the age group of 0–19 years, data were collected from eight municipalities.

Periods of pandemic waves were defined as follows: 1^st^ wave (03/23/2020–05/17/2020/), 2^nd^ wave (06/22/2020–09/27/2020), 3^rd^ wave (10/26/2020–02/21/2021), 4^th^ wave (02/22/2021–06/07/2021), and 5^th^ wave (07/05/2021–09/13/2021).

**Figure 1.**
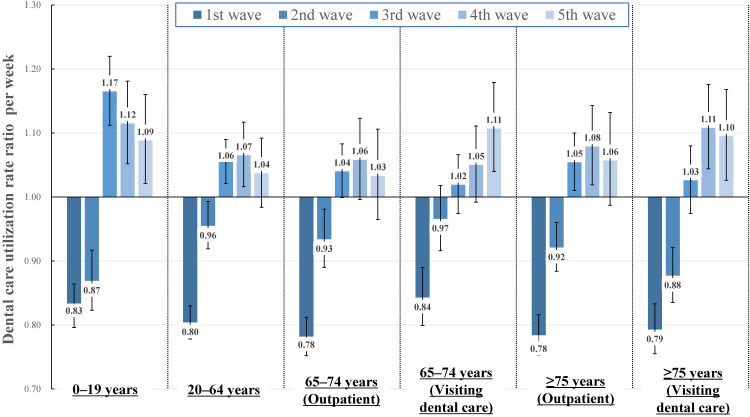
The association between the COVID-19 pandemic and change in dental care utilization rate among nine municipalities. Reference category of estimates is “non-pandemic wave period.” Error bars represent 95% confidence intervals of estimates. For the age group of 0–19 years, data were collected from eight municipalities. The estimates were adjusted for linear temporal trends, holidays per week, and seasonal trends, based on trigonometric functions. Periods of pandemic waves were defined as follows: 1^st^ wave (03/23/2020–05/17/2020), 2^nd^ wave (06/22/2020–09/27/2020), 3^rd^ wave (10/26/2020–02/21/2021), 4^th^ wave (02/22/2021–06/07/2021), and 5^th^ wave (07/05/2021–09/13/2021).

**Figure 2.**
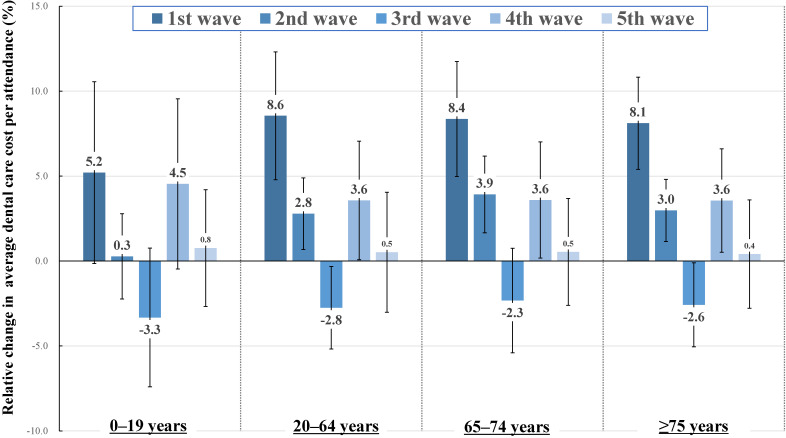
The association between the COVID-19 pandemic and change in average dental care cost per attendance among nine municipalities. Reference category of estimates is “non-pandemic wave period.” Error bars represent 95% confidence intervals of estimates. For the age group of 0–19 years, data were collected from eight municipalities. The estimates were adjusted for linear temporal trends, holidays per week, and seasonal trends, based on trigonometric functions. Periods of pandemic waves were defined as follows: 1^st^ wave (03/23/2020–05/17/2020), 2^nd^ wave (06/22/2020–09/27/2020), 3^rd^ wave (10/26/2020–02/21/2021), 4^th^ wave (02/22/2021–06/07/2021), and 5^th^ wave (07/05/2021–09/13/2021).

Figure 1 shows the results obtained from the fixed-effects model evaluating the association between the COVID-19 pandemic waves and the change in dental care utilization rate with imputed data sets. After adjusting for temporal and seasonal trends, dental care utilization rates significantly declined during the periods of the 1^st^ and 2^nd^ COVID-19 pandemic waves among all age groups and types of dental care utilization. The degree of the decline was larger during the 1^st^ wave than during the 2^nd^ wave. The magnitude of the decline in dental care utilization was larger among 65–74 year and ≥75 years during the 1^st^ wave; however, we did not observe a clear difference between visiting and outpatient dental care. In the 3^rd^ to 5^th^ waves, there was no decrease in dental care utilization compared to before the COVID-19 pandemic. Detailed estimates are presented in eTable 2. In the sensitivity analysis of the complete records, the estimates were similar to those obtained from the imputed datasets (eTable 3). Figure 2 shows the relative change in the average dental care cost per attendance during the COVID-19 pandemic waves. During the 1^st^ wave of the COVID-19 pandemic, the average dental care cost per attendance increased significantly in all age groups. Especially among 20–64 years, 65–74 years, and ≥75 years, the average dental care cost per attendance increased by approximately 8.0%. After the 1^st^ wave, although the average dental care cost per attendance fluctuated, the proportion of fluctuations was considerably small when compared to the 1^st^ wave. Detailed estimates of absolute and relative changes in the average dental care cost per attendance are presented in eTable 4. In the sensitivity analysis of the complete records, the estimates were similar to those obtained from the imputed data sets (eTable 5).

## DISCUSSION

### Summary of the main results

The present time-series study among nine municipalities in Japan showed that dental care utilization significantly decreased during the 1^st^ and 2^nd^ waves of the COVID-19 pandemic, irrespective of age group or type of dental care utilization. In addition, the magnitude of decline in the 1^st^ wave was dominant among older adults (65–74 years and ≥75 years). The decline in dental care utilization gradually recovered during subsequent pandemic waves. In contrast, the average dental care cost per attendance significantly increased during the 1^st^ pandemic wave, and the magnitude was higher among 20–64 years, 65–74 years, and ≥75 years with an increase of approximately 8.0%. In the waves after the 1^st^ wave, although there were significant fluctuations in the average dental care cost per attendance among all age groups, the magnitudes were smaller than in the 1^st^ pandemic wave.

### Possible explanation of the present findings

In this study, we found a large decline in dental care utilization across all age groups during the initial wave of the COVID-19 pandemic. Similar changes in dental care utilization during the lockdown or emergency state under the COVID-19 pandemic were also reported in the previous studies conducted in Japan,^10^^,^^19^ the United States,^6^^,^^8^ and Canada.^9^ They also reported that the decline in dental care utilization immediately recovered to the previous level. However, our results showed that even during the 2^nd^ wave of the COVID-19 pandemic, significant declines in dental care utilization were observed among all age groups. This contradiction is considered to be due to the modeling used in our study. Most of the previous studies were descriptive studies and only compared the descriptive statistics (ie, crude rate) or visualized them as a graph; whereas, we considered the fluctuation of dental care utilization due to seasonal trends and holidays in the present study. Therefore, we could eliminate the influence of other factors and evaluate the impact on dental care utilization during subsequent pandemic waves. The decline in dental care utilization gradually increased after the initial wave. Although a previous study reported a decline in the initial COVID-19 pandemic wave among those with employee insurance in Japan, which mainly included those aged <75 years employed by the company and their families, we observed similar trends among the relatively older population in this study.

Possible mechanisms underlying the significant decrease in dental care utilization during 1st and 2nd waves of COVID-19 can be explained as follows: The Japanese government declared 1st state of emergency in the 1st wave of the COVID-19 pandemic, which discourage any unnecessary leaving home, including daytime.^20^ Although the state did not have legal binding, it had affected people activity, including dental care attendance. States of emergency were also declared in the 3rd and 4th wave; however, these states were mainly focused on restricting leaving home at night, which would have less impact on daytime activity. Therefore, a decline in dental care utilization was not observed in 3rd and 4th waves of the COVID-19 pandemic. In addition, the proportion of those avoiding going out has gradually decreased in Japan since the 1st wave of the COVID-19 pandemic.^21^ The medical care provision system was also strengthened from the 1st wave.^20^ Therefore, dental care utilization was largely decreased in 1^st^ wave and mitigated in more current waves, although we observed a decline in dental care utilization remaining during 2^nd^ wave. The change in people’s activity patterns, the different focus of the state of emergency, and the strengthening of the medical care provision system would have influenced recovery from the decline in dental care utilization. Our study showed that the decline in dental care utilization was higher among older adults aged ≥65 years. A previous study reported that physician visits of patients with chronic diseases decreased during the 1^st^ COVID-19 wave in Japan.^4^ Another study also showed that older adults tended to avoid social contact during the COVID-19 pandemic.^21^ Therefore, older adults who were highly comorbid with chronic diseases could also have avoided dental care utilization.

Regarding the dental care cost per attendance, we observed a large increase during the 1^st^ pandemic wave across all age groups. A previous descriptive study that investigated the dental care cost during the state of emergency among Japanese employees and their families aged <75 years reported the cost increment during the COVID-19 pandemic wave.^10^ We also confirmed similar results in this study, including among all age groups. A previous study also suggested that the increase in dental care costs per attendance prolonged the subsequent pandemic waves,^10^ and the descriptive statistics of the present study also showed an increase in dental care costs in the descriptive statistics of the subsequent pandemic waves. However, the statistical model considering temporal and seasonal fluctuations and the influence of holidays suggested that the large increase in dental care cost per attendance was limited to the 1^st^ wave of the COVID-19 pandemic. We also observed small fluctuations in dental care cost per attendance through the 2nd to 4th waves. Although these fluctuations were smaller than those in 1st wave, they were observed across all age groups. These fluctuations suggest the prolonged influence of the COVID-19 pandemic on dental care utilization, and future research evaluating more detailed dental provision patterns is desired. In the descriptive statistics, the average per-attendance care cost in 3^rd^ wave was higher than in the other waves, whereas in the statistical model, such an increase was not observed. The period of 3^rd^ wave included December and January, when the number of days of clinic closure was high owing to holidays. In Japan, additional costs are incurred when clinics provide treatment to patients on holidays. In such cases, the patient’s motive for visiting the clinic is also considered as an emergency, which requires a more expensive procedure than routine oral care. Therefore, a higher average per-attendance cost was observed only in the descriptive statistics without considering holidays.

In this study, we found that during the 1^st^ pandemic wave, there was a decrease in dental care utilization and an increase in dental care costs per utilization. This phenomenon could be explained by the fact that during the 1^st^ pandemic wave, patients and dentists avoided non-urgent dental care or dentists increased the intensity of care per attendance to decrease the frequency of dental visits.^10^ A previous study reported that the decrease in dental care was larger among non-urgent dental care or dental check-ups compared to urgent dental care or dental visits due to tooth pain.^6^^,^^22^ For urgent dental care attendance in Japan, the patients have to pay more charges, including the fee for a patient’s first visit, extra additional charges, and the cost of testing, such as radiography, than continuous dental care. These medical care fee systems also contributed to an increase in dental care cost per attendance during 1st wave of the COVID-19 pandemic. In addition, the medical and dental fee for reimbursement was revised in April 2020. The revision may also have affected the changes in dental care cost per attendance. The change in dental care costs got smaller through the 1st to 5th waves of the COVID-19 pandemic, and the increase in dental care costs was not prolonged for more current waves. However, the revision rate of the reimbursement on the change in dental care costs in 2020 was 0.59%,^10^ and the fluctuation of dental care costs during the COVID-19 pandemic waves could be partially explained by the 2020 revision in dental care costs. Future longitudinal research investigating the changes in the pattern of dental care during the COVID-19 pandemic waves would contribute to revealing the underlying mechanism.

### Implications

From the public health perspective, oral diseases, including dental caries, periodontal diseases, and tooth loss, are highly prevalent globally.^23^ Although an initial report highlighted the risk of SARS-CoV-2 transmission in dental settings,^5^ recent studies have reported that the actual risk of SARS-CoV-2 transmission is considerably low.^24^^,^^25^ There may have been no scientific reason for patients to refrain from necessary dental attendance because of COVID-19. The government and dental associations need to provide patients with up-to-date and relevant evidence-based information on dental attendance during the pandemic periods based on scientific evidence.

### Limitations and strengths

The present study has some limitations. First, our study included those living in nine municipalities in Japan; therefore, the characteristics of the population in our study may have differed from those of the general population in Japan. However, the municipalities included in the present study were located across Japan and the population varied; therefore, the characteristics of the study population were not considered to differ significantly from those of the general Japanese population. Second, although we used health claims data from the National Health Insurance, some of the visiting dental care was also provided by long-term care insurance.^14^ Therefore, the number of dental care services provided may have been higher than estimated. However, dental care providers are usually the same clinics or dentists as those providing national health insurance services. Therefore, the changing trend in the COVID-19 pandemic wave would be similar, even if visiting dental care based on long-term care services was included. Third, although we estimated the insured population based on government statistics and imputed between the time-point linearly, it may differ from the actual insured population of each municipality due to fluctuation of enrollment and withdrawal. The enrollment and withdrawal were considered to be in equilibrium with these trends. We also considered seasonal fluctuations in the statistical model; therefore, the bias due to employing an imputed number of insured populations was small.

In contrast, the present study also has several strengths. First, we used the individual records of health claims data, which enabled us to create weekly data and analyze it by age group and type of dental care utilization, which provided more detailed findings. Second, previous studies used data only from employee and their family aged <75 years, and they did not evaluate the influence of the COVID-19 pandemic on dental care utilization among older adults aged ≥75 years, who were more susceptible to COVID-19. In contrast, the population of this study included the data of older adults aged ≥75 years and covered whole age groups. Therefore, we could provide comprehensive insights into the influence of the COVID-19 pandemic on dental care utilization in the Japanese population. Third, we employed the fixed-effects model, allowing us to eliminate confounding due to time-invariant covariates while evaluating the association between the COVID-19 pandemic and dental care utilization. However, there is the possibility that the influence of COVID-19 pandemic waves differed by municipality. Future studies using a random-effects model that considers a random slope with a larger number of municipalities could solve these problems.

### Conclusion

This study evaluated the change in dental care utilization during the COVID-19 pandemic waves in Japan and showed that dental care utilization decreased during the 1^st^ wave, especially in older adults, irrespective of outpatient or visiting dental care. In addition, the decrease recovered, but was still significant until the 2^nd^ wave. In contrast, the dental care cost per attendance increased during the 1^st^ wave. This phenomenon may reflect changes in the patterns of dental care provision to patients. A system that provides continuous dental care even during a pandemic of highly infectious diseases is required to maintain people’s health.

## SUPPLEMENTARY MATERIAL

eTable 1. The definition of the procedure codes for dental care utilization

eFigure 1. Daily incidence of COVID-19 in Japan and the COVID-19 pandemic periods of the present study. The non-pandemic wave periods is presented as the periods painted with the following color . Data was obtained from https://www.mhlw.go.jp/stf/covid-19/open-data.html. COVID-19, coronavirus disease 2019.

eFigure 2. The insured population of the National Health Insurance among the 0–19-year age group by municipality

eFigure 3. The insured population of the National Health Insurance among the 20–64-year age group by municipality

eFigure 4. The insured population of the National Health Insurance among the 65–74-year age group by municipality

eFigure 5. The insured population of the Latter-Stage Elderly Healthcare System among the ≥75-year age group by municipality

eFigure 6. The periods without healthcare incurrence claim data by municipality. For the ≥75-year age groups of “city I”, the healthcare incurrence claim data were missing between 113 and 143 epidemiological weeks.

eFigure 7. Total dental care utilization rate per week among the 0–19-year age group by municipality. For “city E”, no healthcare incurrence claim data were included among the 0–19-year age group.

eFigure 8. Total dental care utilization rate per week among the 20–64-year age group by municipality

eFigure 9. Outpatient dental care utilization rate per week among the 65–74-year age group by municipality

eFigure 10. Visiting dental care utilization rate per week among the 65–74-year age group by municipality

eFigure 11. Outpatient dental care utilization rate per week among the ≥75-year age group by municipality

eFigure 12. Visting dental care utilization rate per week among the ≥75-year age group by municipality

eFigure 13. The average dental care cost per attendance among the 0–19-year age group by municipality. For “city E”, no healthcare incurrence claim data were included among 0–19-year age group.

eFigure 14. The average dental care cost per attendance among the 20–64-year age group by municipality

eFigure 15. The average dental care cost per attendance among the 65–74-year age group by municipality

eFigure 16. The average dental care cost per attendance among the ≥75-year age group by municipality

eTable 2. The association between the COVID-19 pandemic and change in dental care utilization rate per week among nine municipalities with imputed datasets

eTable 3. The association between the COVID-19 pandemic and change in dental care utilization rate per week among nine municipalities with complete records

eTable 4. The association between the COVID-19 pandemic and change in average dental care cost per attendance among nine municipalities with imputed datasets

eTable 5. The association between the COVID-19 pandemic and change in average dental care cost per attendance among nine municipalities with complete records
