# Supplementary material for: Changes in the Utilization of Outpatient and Visiting Dental Care and Per-attendance Care Cost by Age Groups During COVID-19 Pandemic Waves in Japan: A Time-series Analysis From the LIFE Study
Source: J Epidemiol. 2024 Nov 5;34(11):553–9. doi: 10.2188/jea.JE20230323 (PMC11464850; doi:10.2188/jea.JE20230323)
Supplement: Supplementary file 1 [file je-34-553-s001.pdf]

eTable 1. The definition of the procedure codes for dental care utilization

Outpatient dental care	301000110, 301000210, 301001610, 301001710
Home-visit dental care	303000110, 303006550, 303008750, 303008850, 303009710, 303009810, 303000210, 303006650, 303008850, 303008950, 303009050, 303004610, 303009150, 303006750, 303009250, 303001910, 303002010, 303007310, 303007410, 303007510, 303006310, 303006410, 303006250

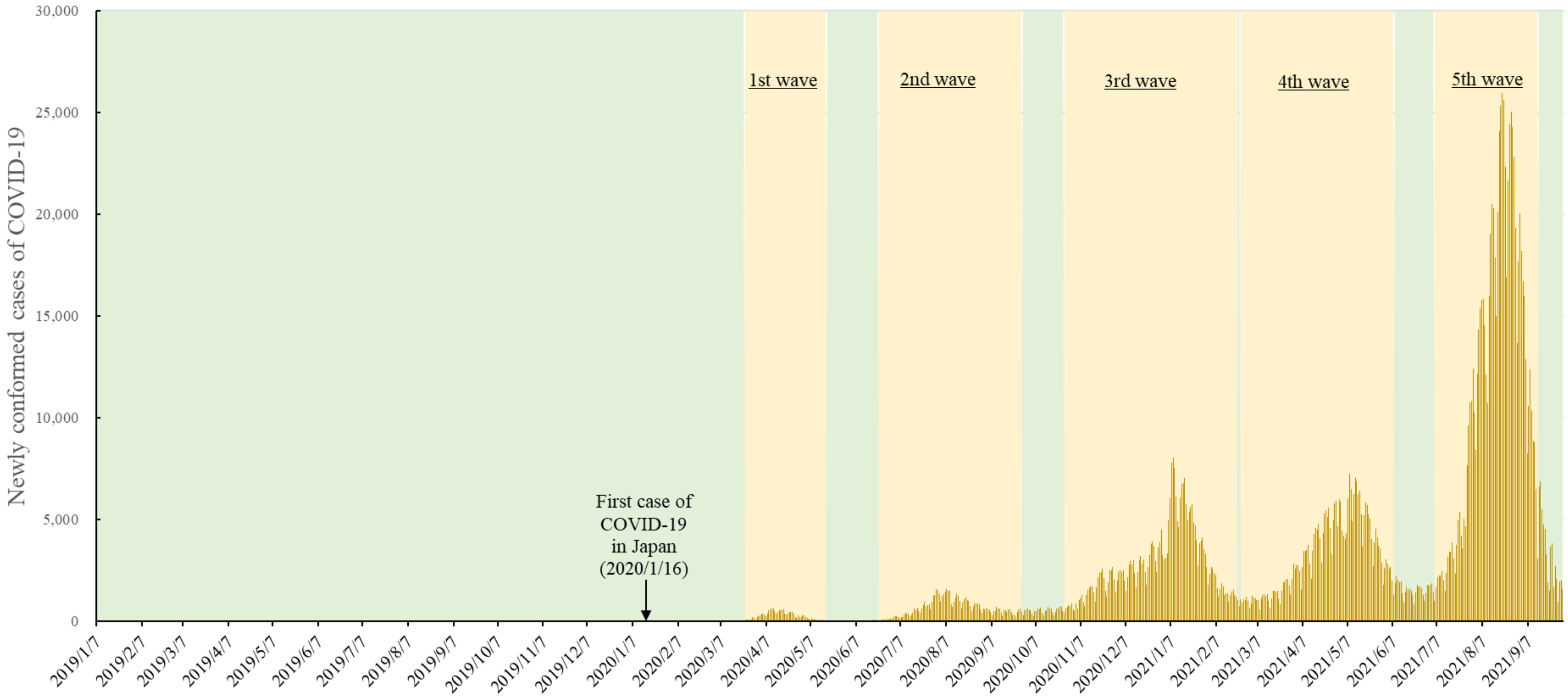

eFigure 1. Daily incidence of COVID-19 in Japan and the COVID-19 pandemic periods of the present study. The non-pandemic wave periods is presented as the periods painted with the following color . Data was obtained from https://www.mhlw.go.jp/stf/covid-19/open-data.html. COVID-19, coronavirus disease 2019.

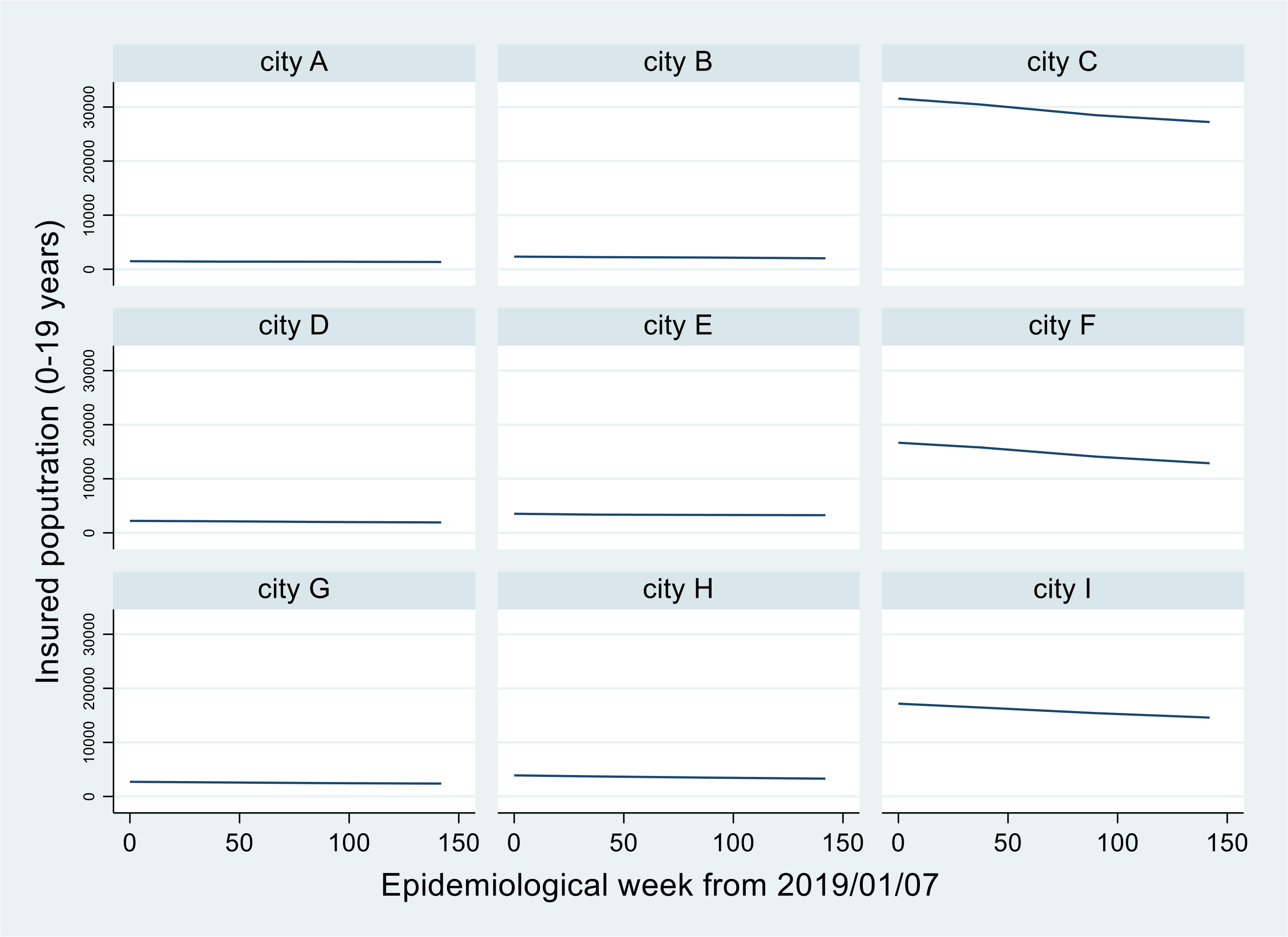

eFigure 2. The insured population of the National Health Insurance among the 0–19-year age group by municipality

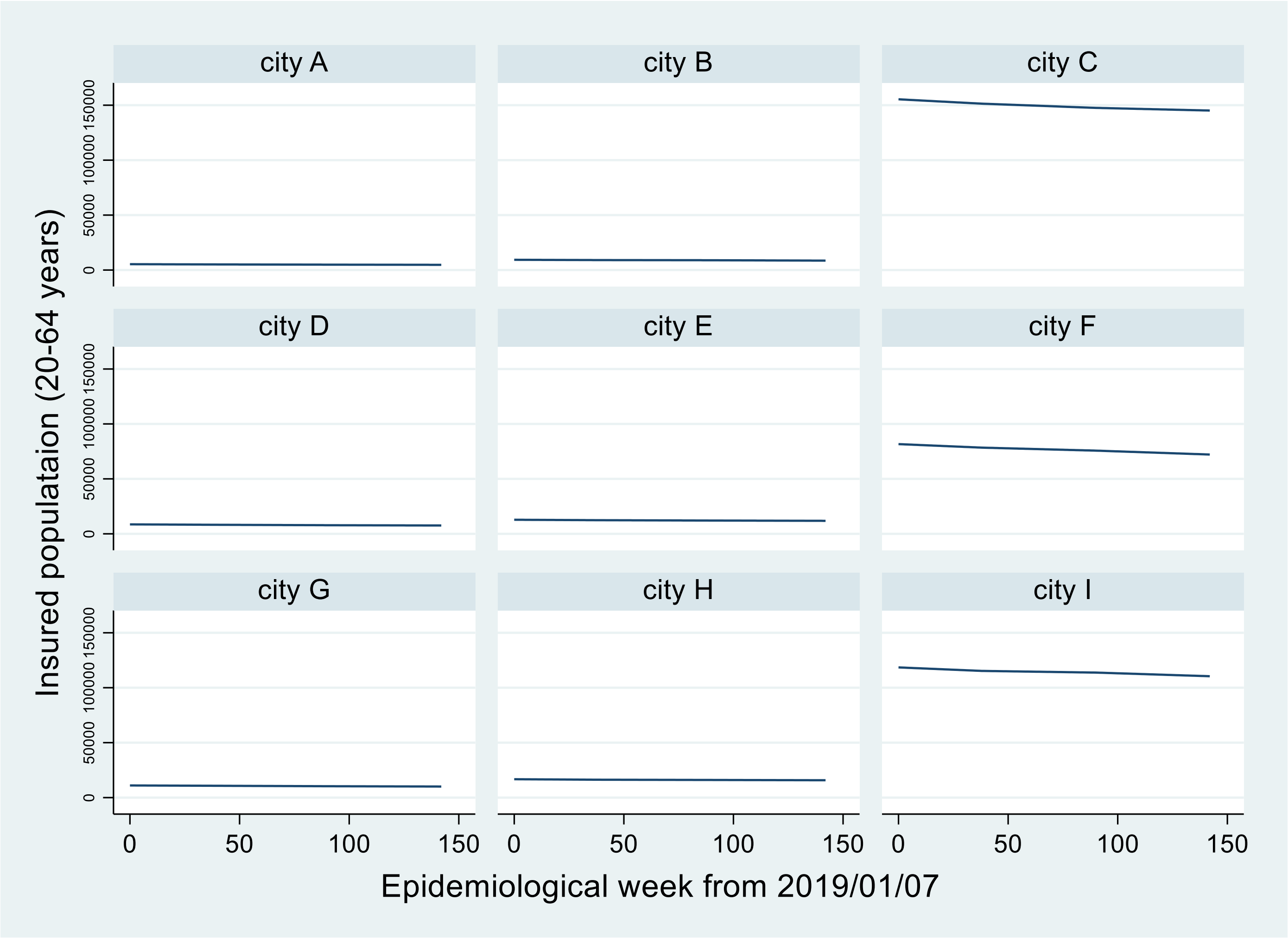

eFigure 3. The insured population of the National Health Insurance among the 20–64-year age group by municipality

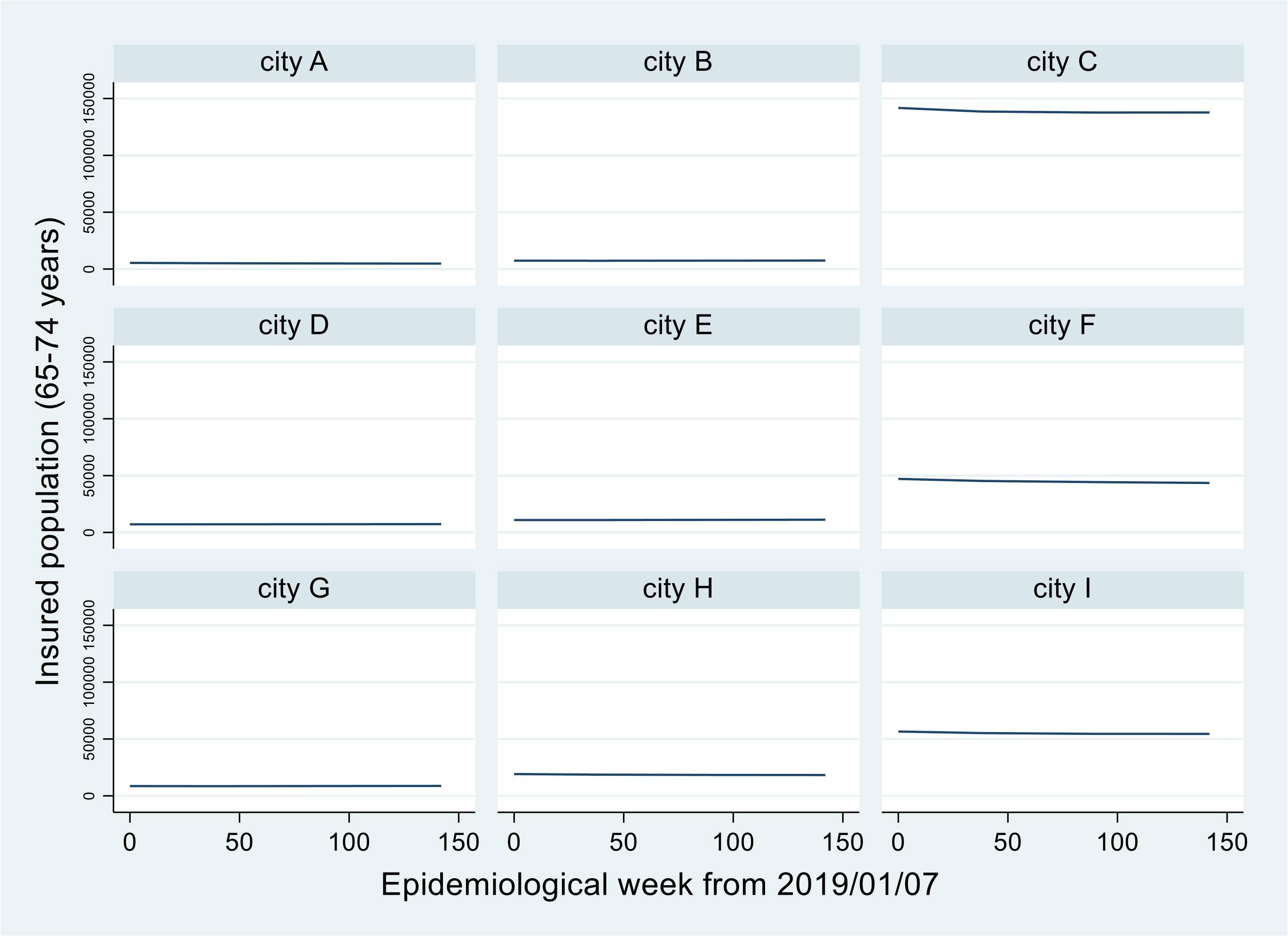

eFigure 4. The insured population of the National Health Insurance among the 65–74-year age group by municipality

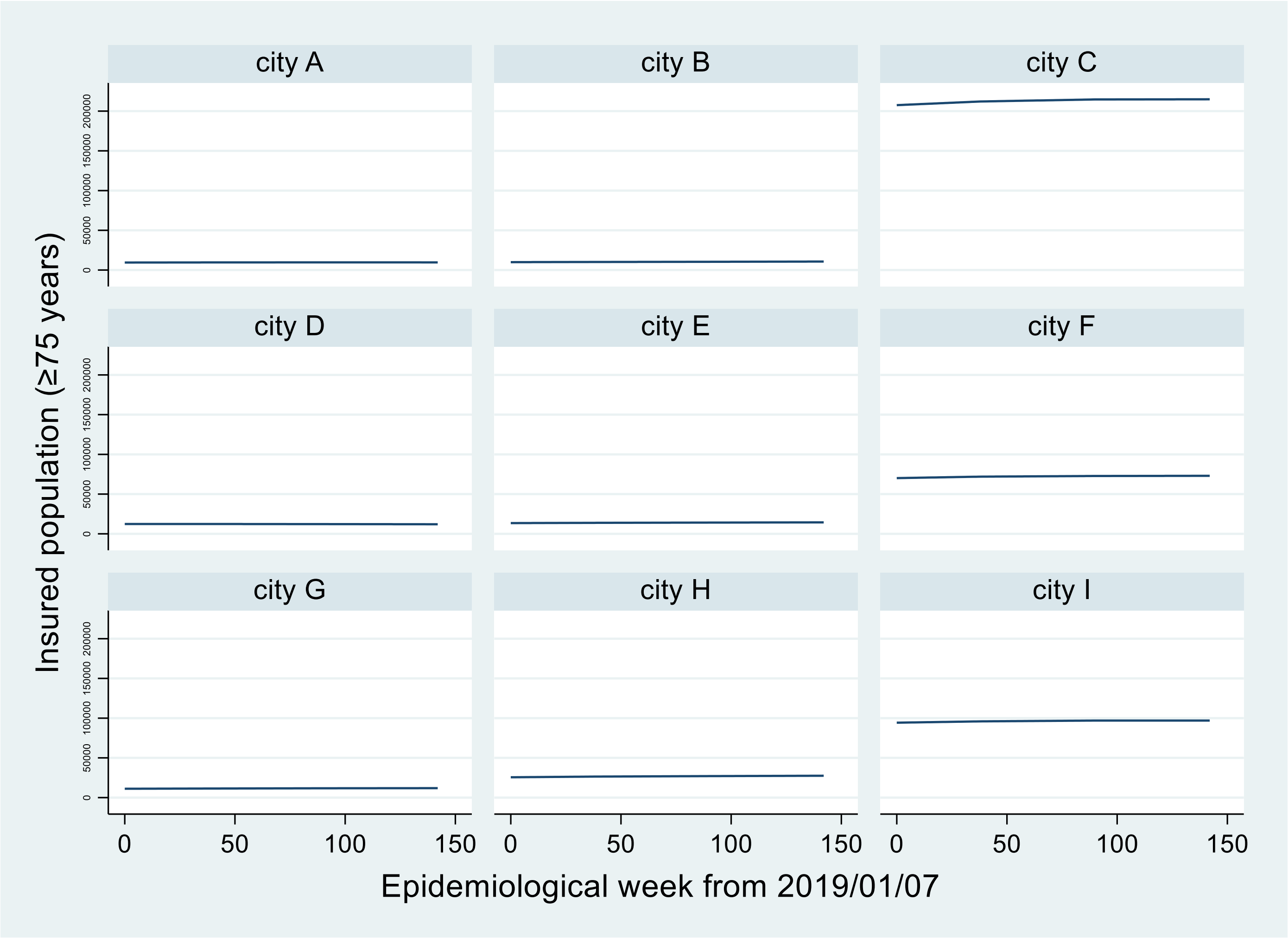

eFigure 5. The insured population of the Latter-Stage Elderly Healthcare System among the ≥75-year age group by municipality

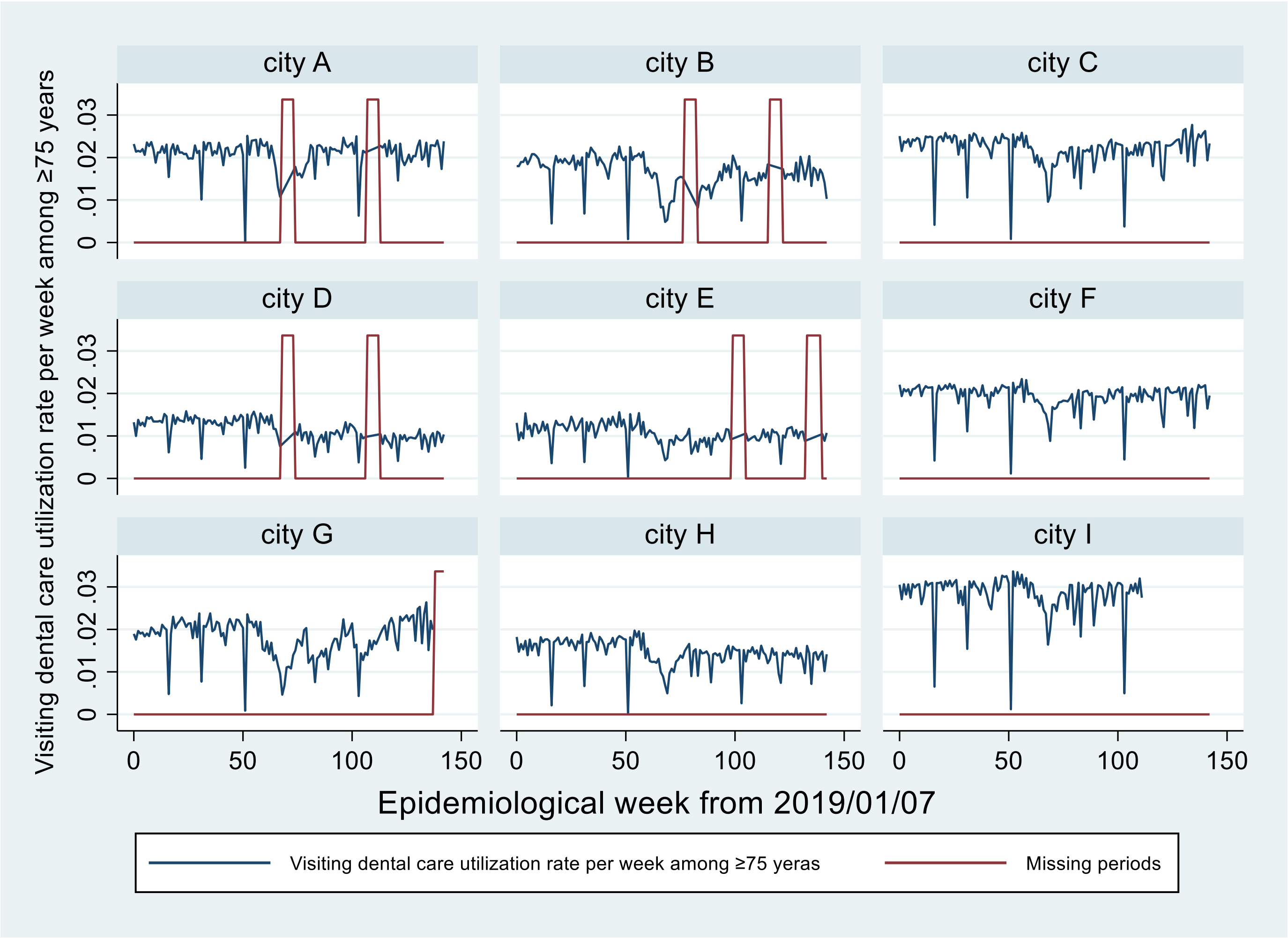

eFigure 6. The periods without healthcare incurrence claim data by municipality. For the ≥75-year age groups of "city I", the healthcare incurrence claim data were missing between 113 and 143 epidemiological weeks.

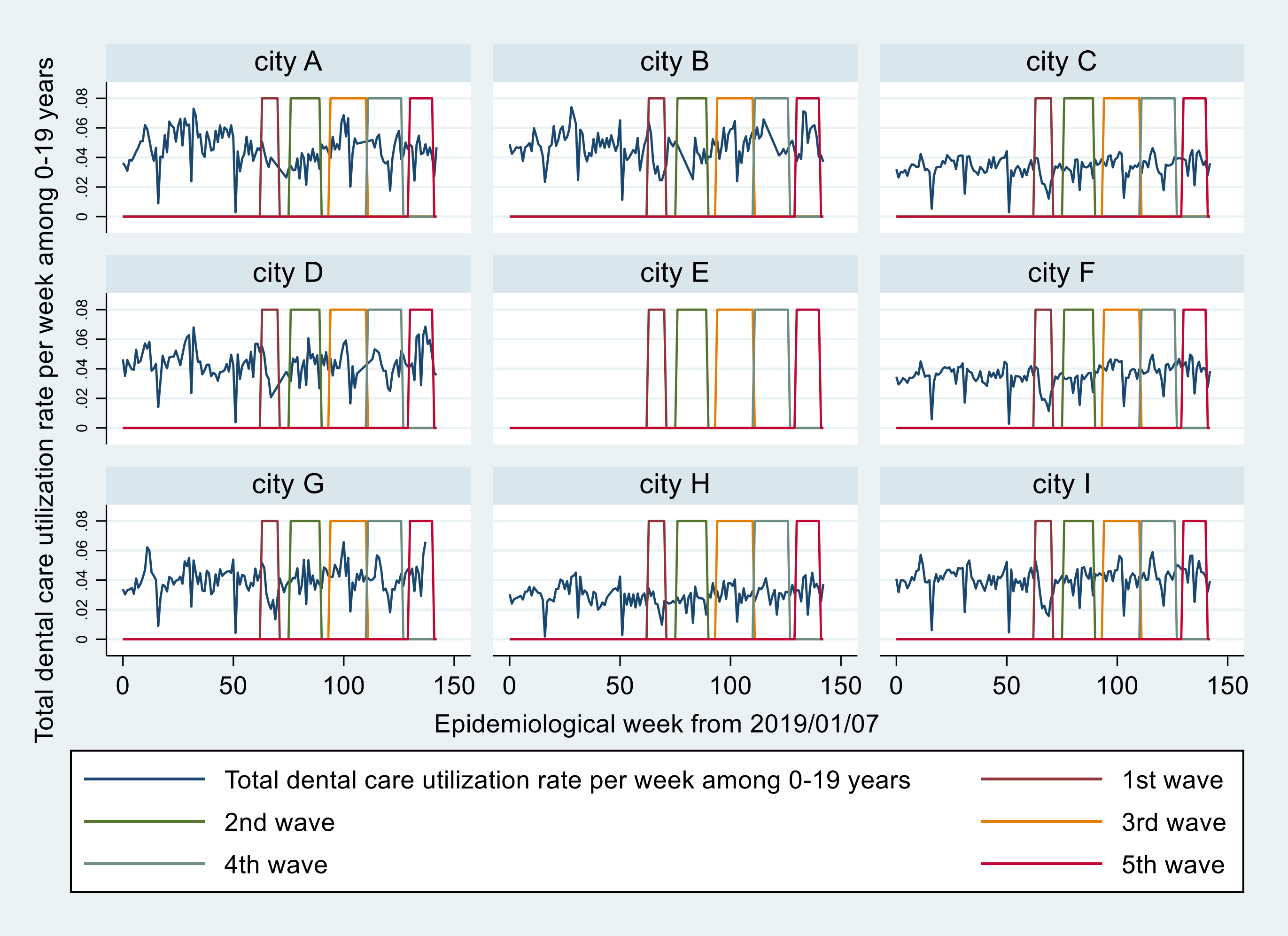

eFigure 7. Total dental care utilization rate per week among the 0–19-year age group by municipality. For "city E", no healthcare incurrence claim data were included among the 0–19-year age group.

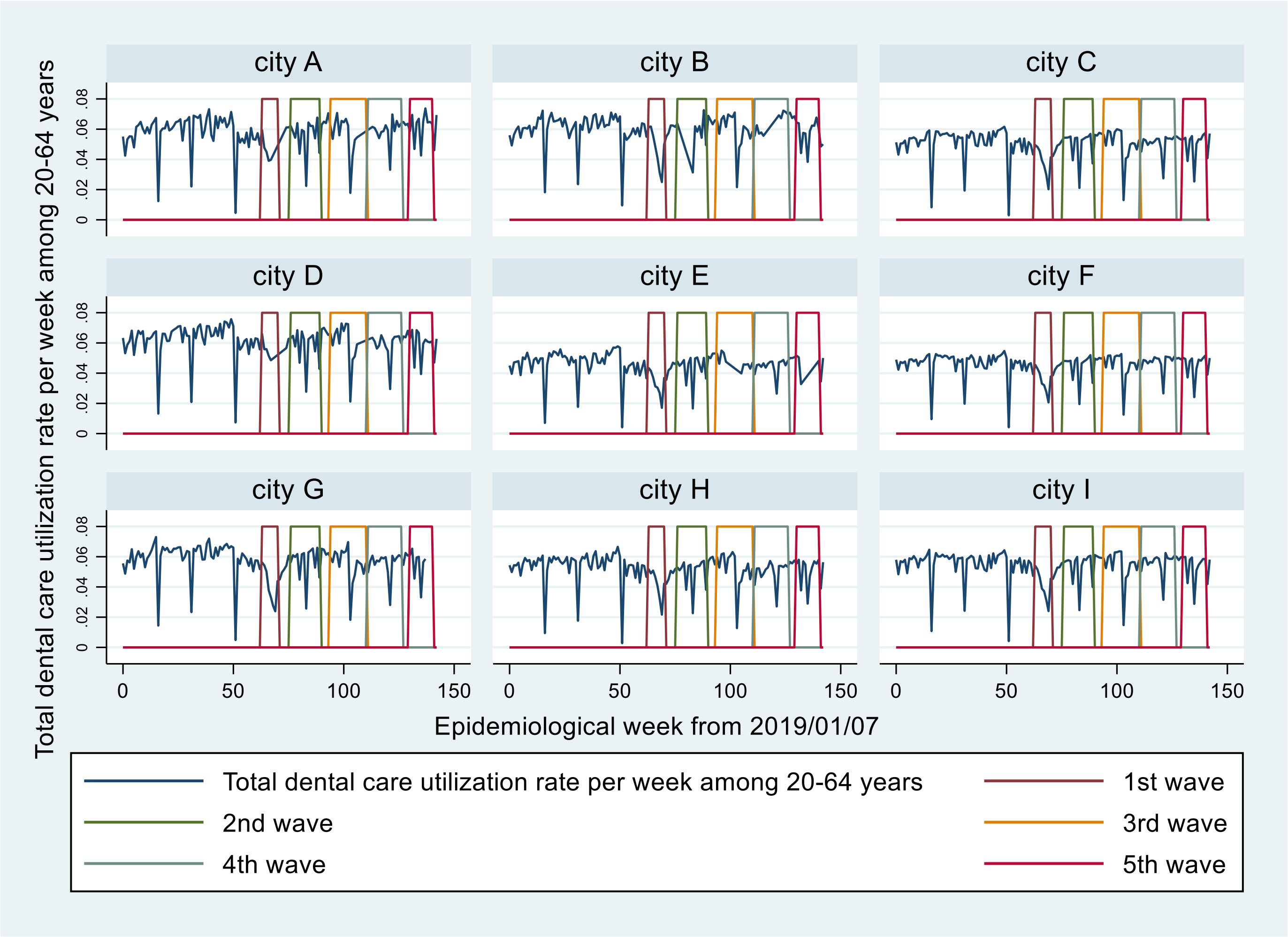

eFigure 8. Total dental care utilization rate per week among the 20–64-year age group by municipality

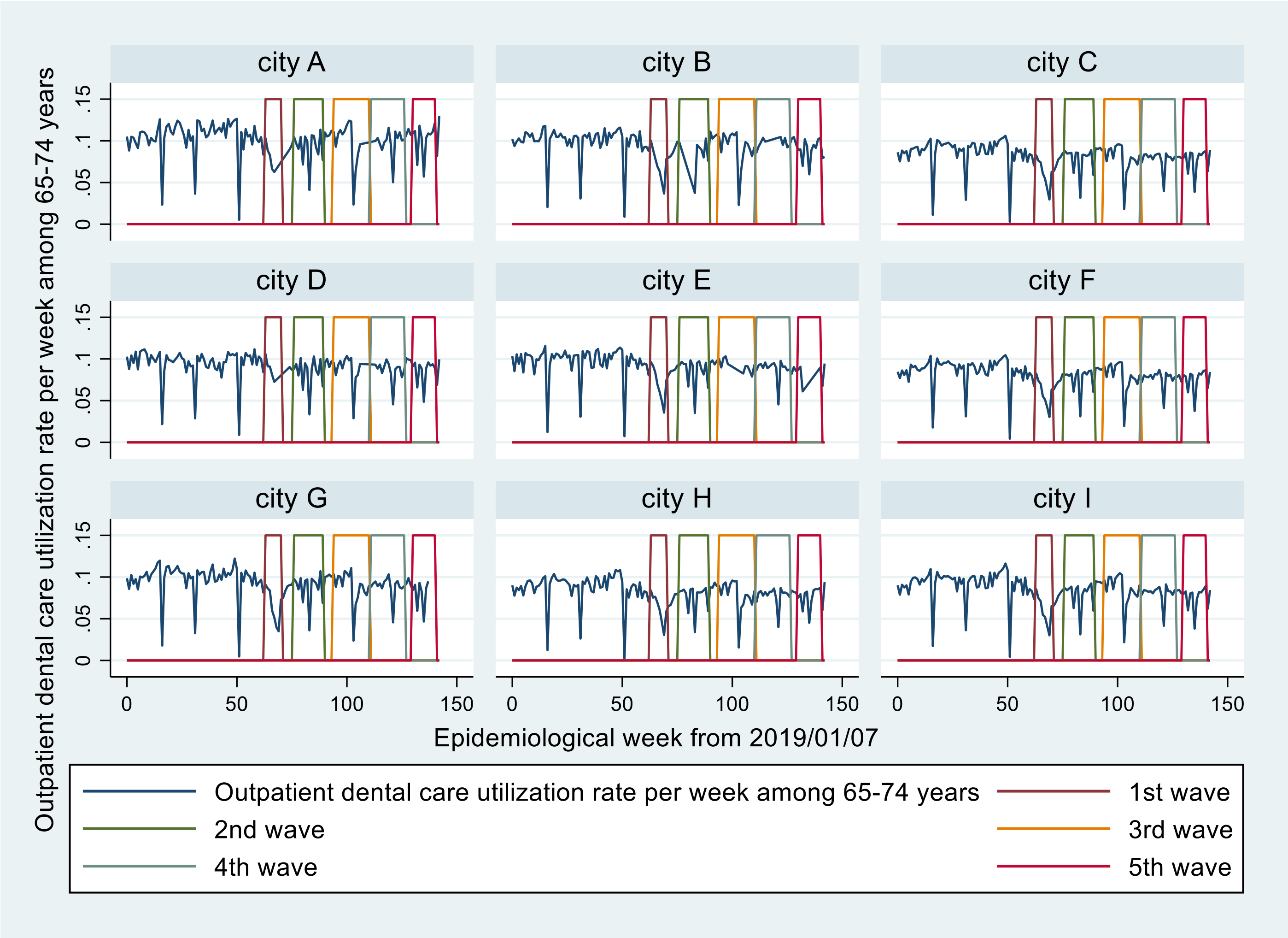

eFigure 9. Outpatient dental care utilization rate per week among the 65–74-year age group by municipality

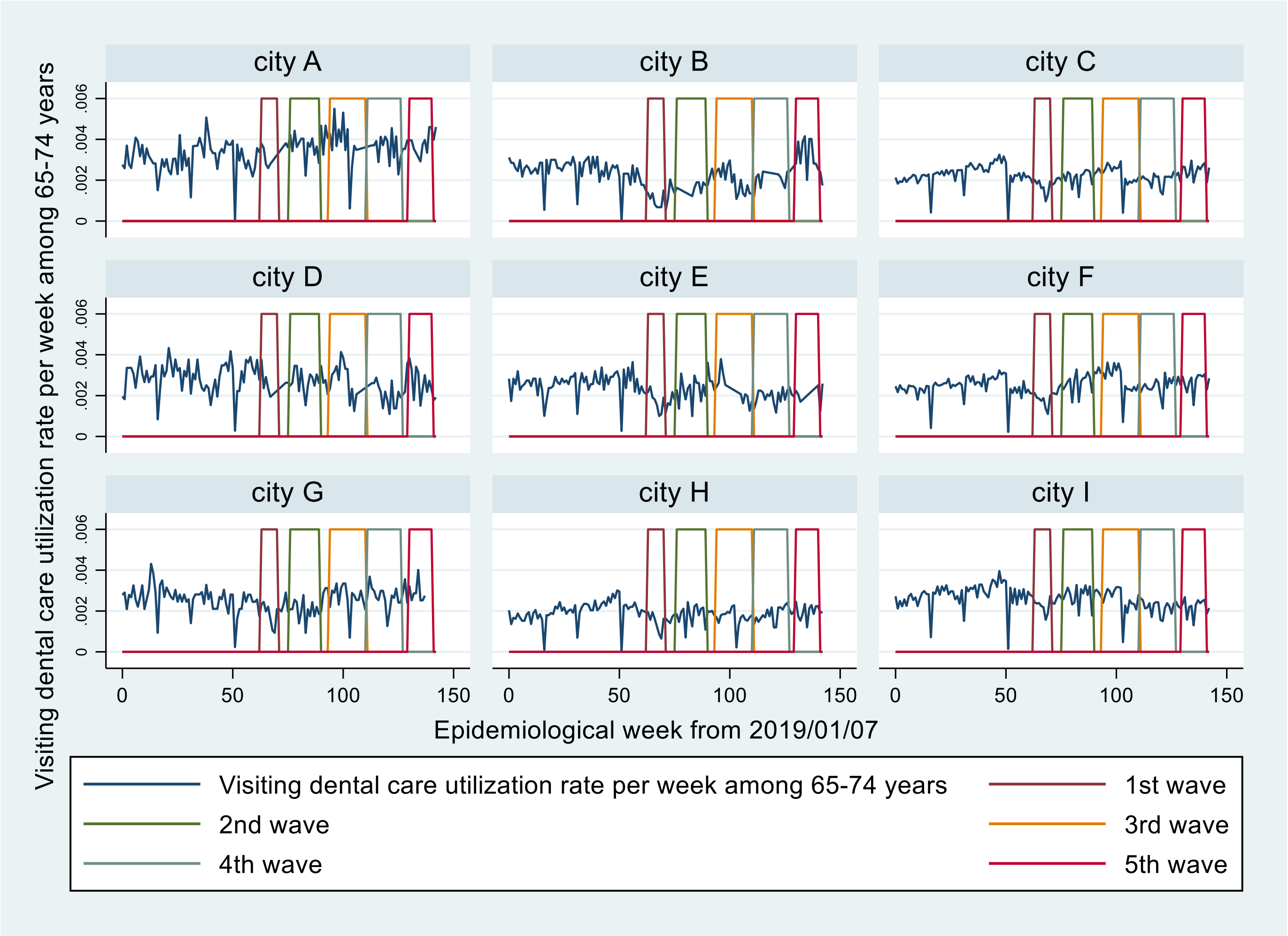

eFigure 10. Visiting dental care utilization rate per week among the 65–74-year age group by municipality

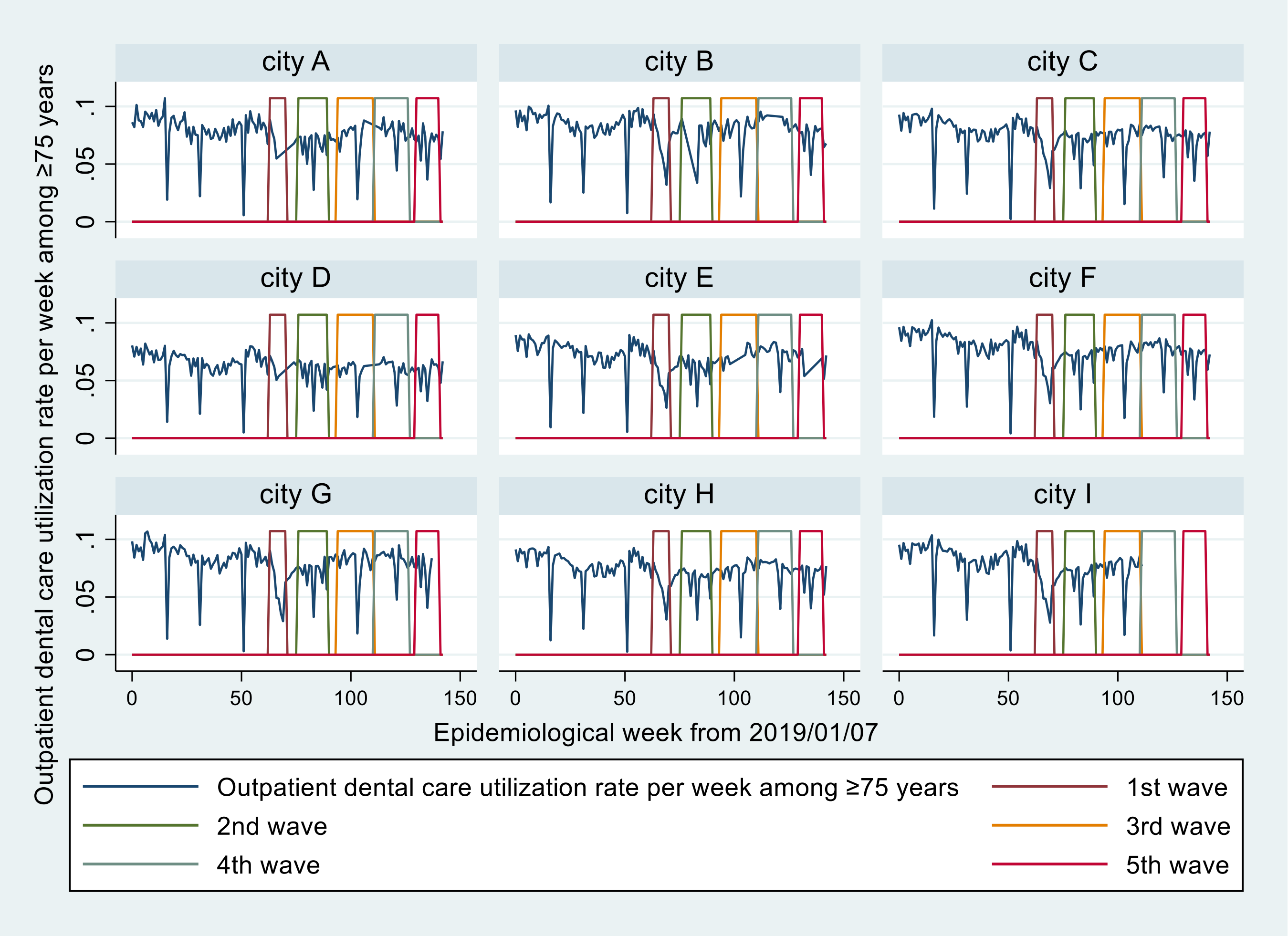

eFigure 11. Outpatient dental care utilization rate per week among the ≥75-year age group by municipality

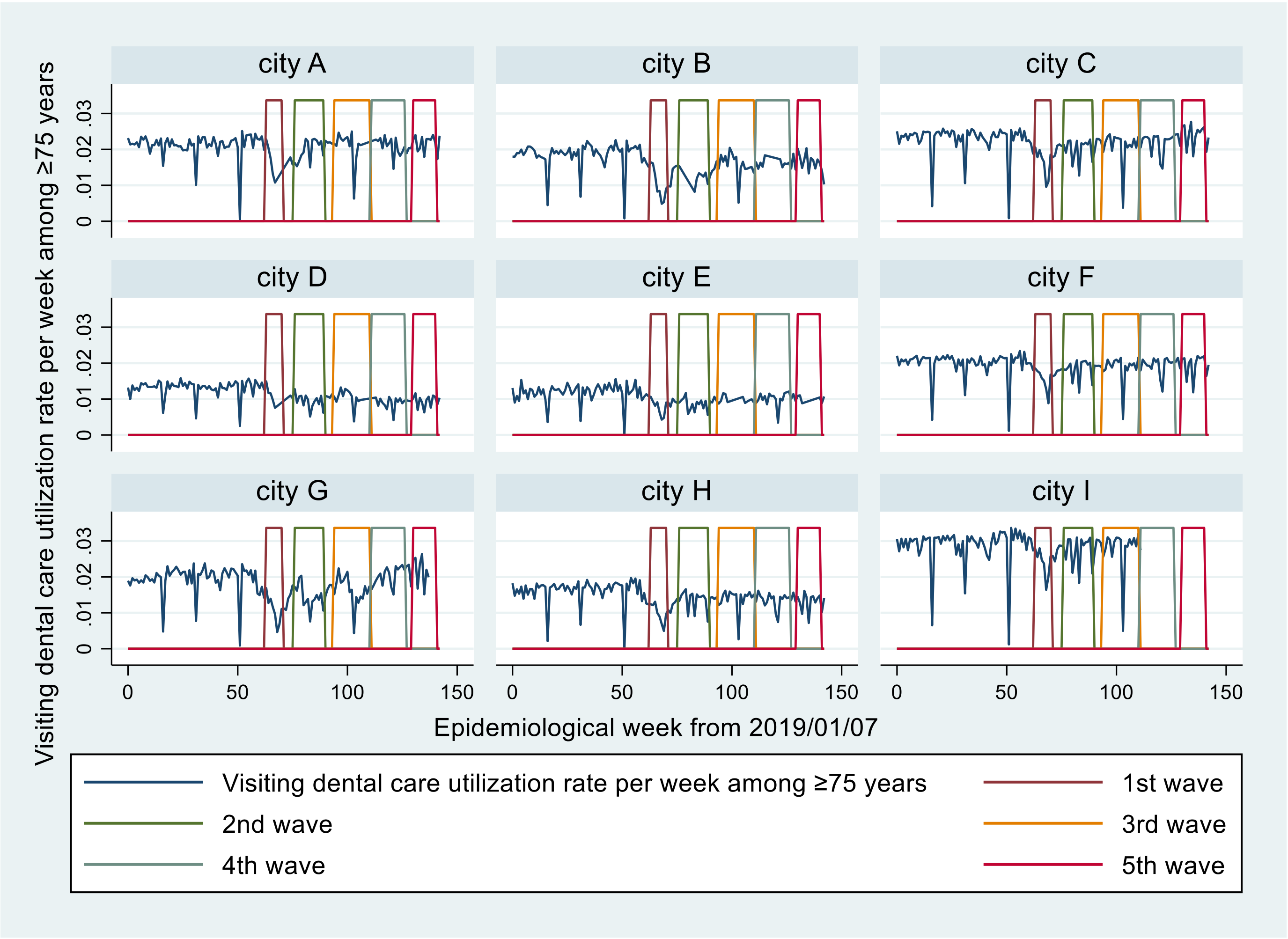

eFigure 12. Visting dental care utilization rate per week among the ≥75-year age group by municipality

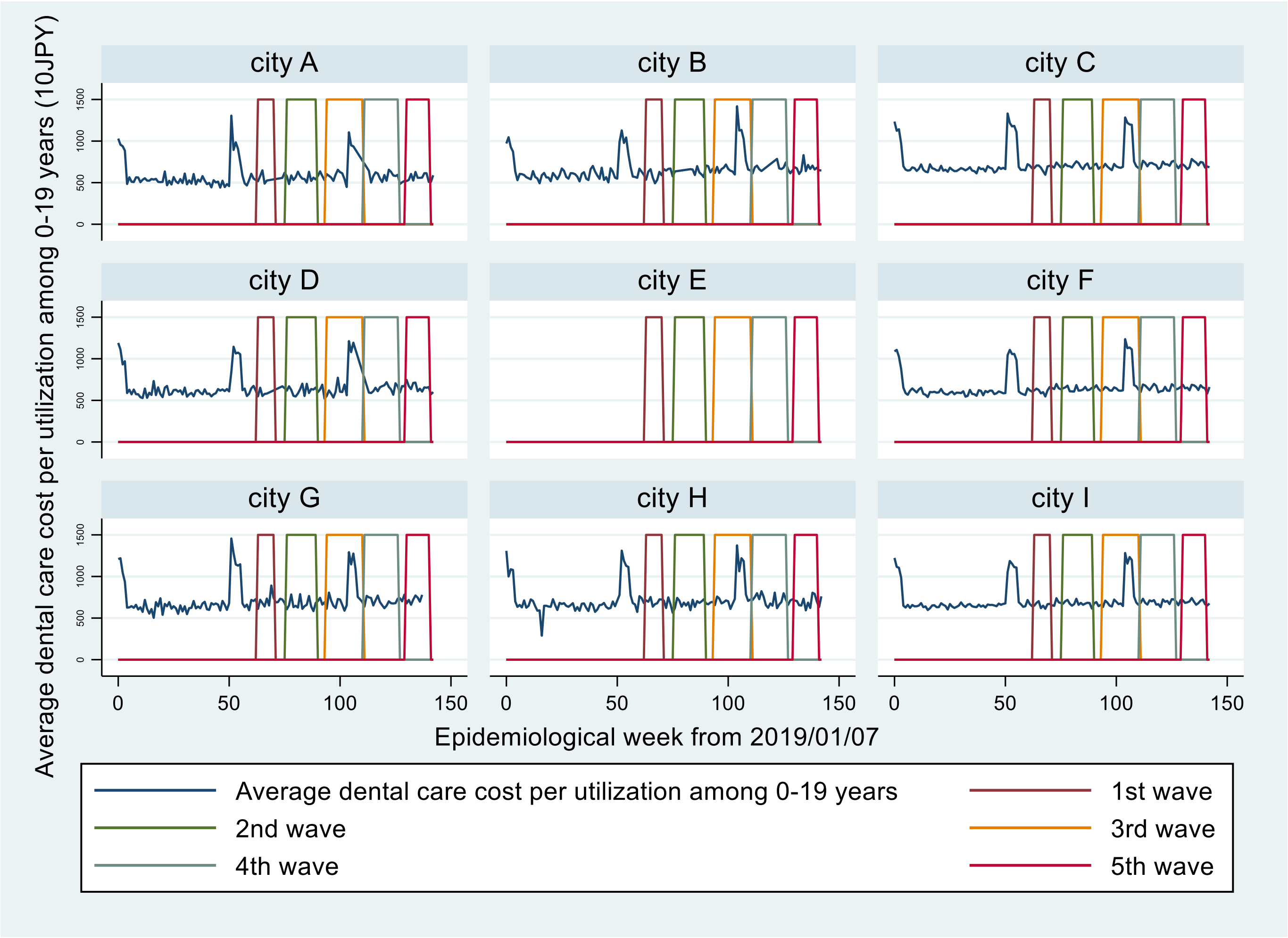

eFigure 13. The average dental care cost per attendance among the 0–19-year age group by municipality. For "city E", no healthcare incurrence claim data were included among 0–19-year age group.

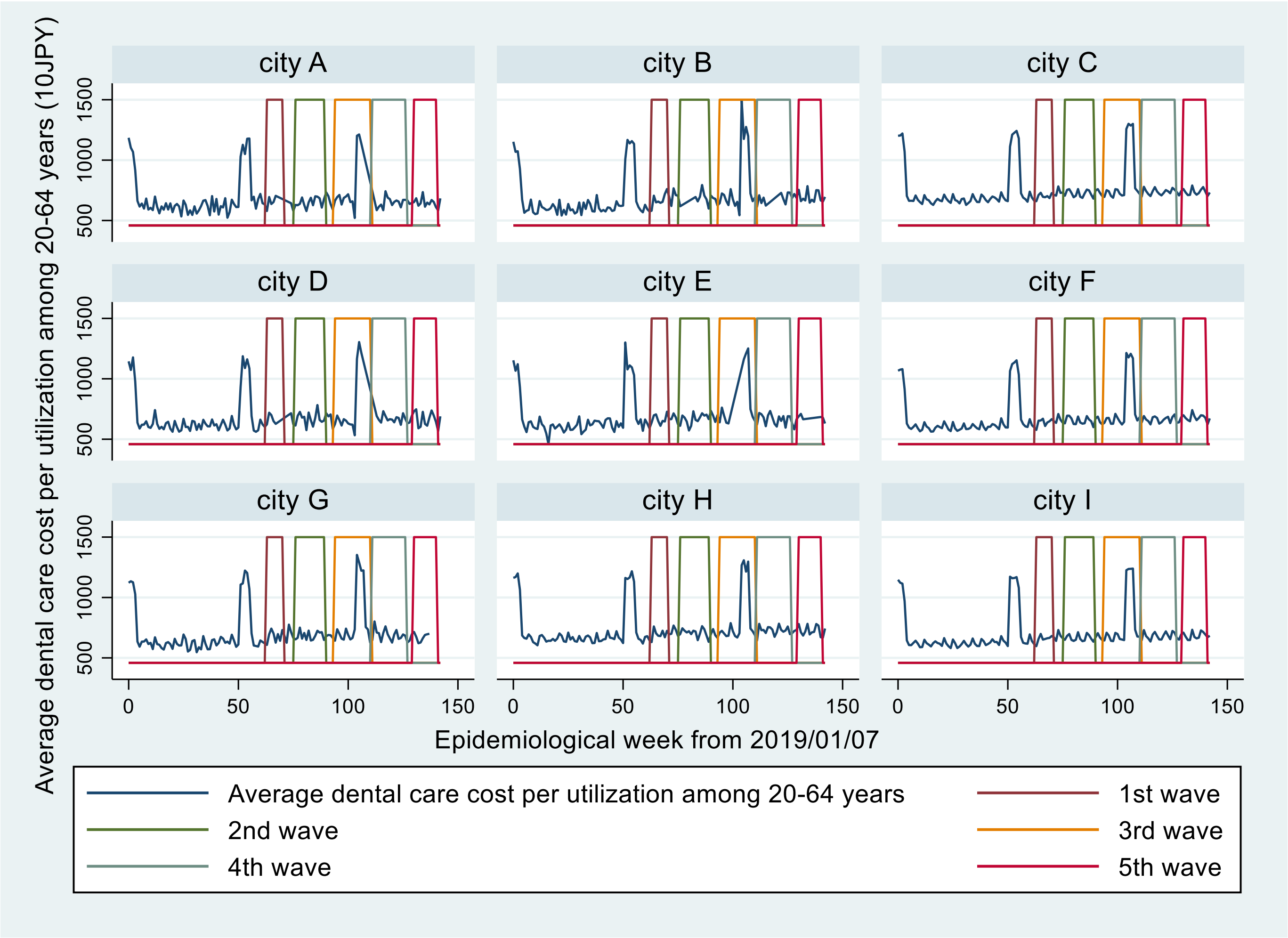

eFigure 14. The average dental care cost per attendance among the 20–64-year age group by municipality

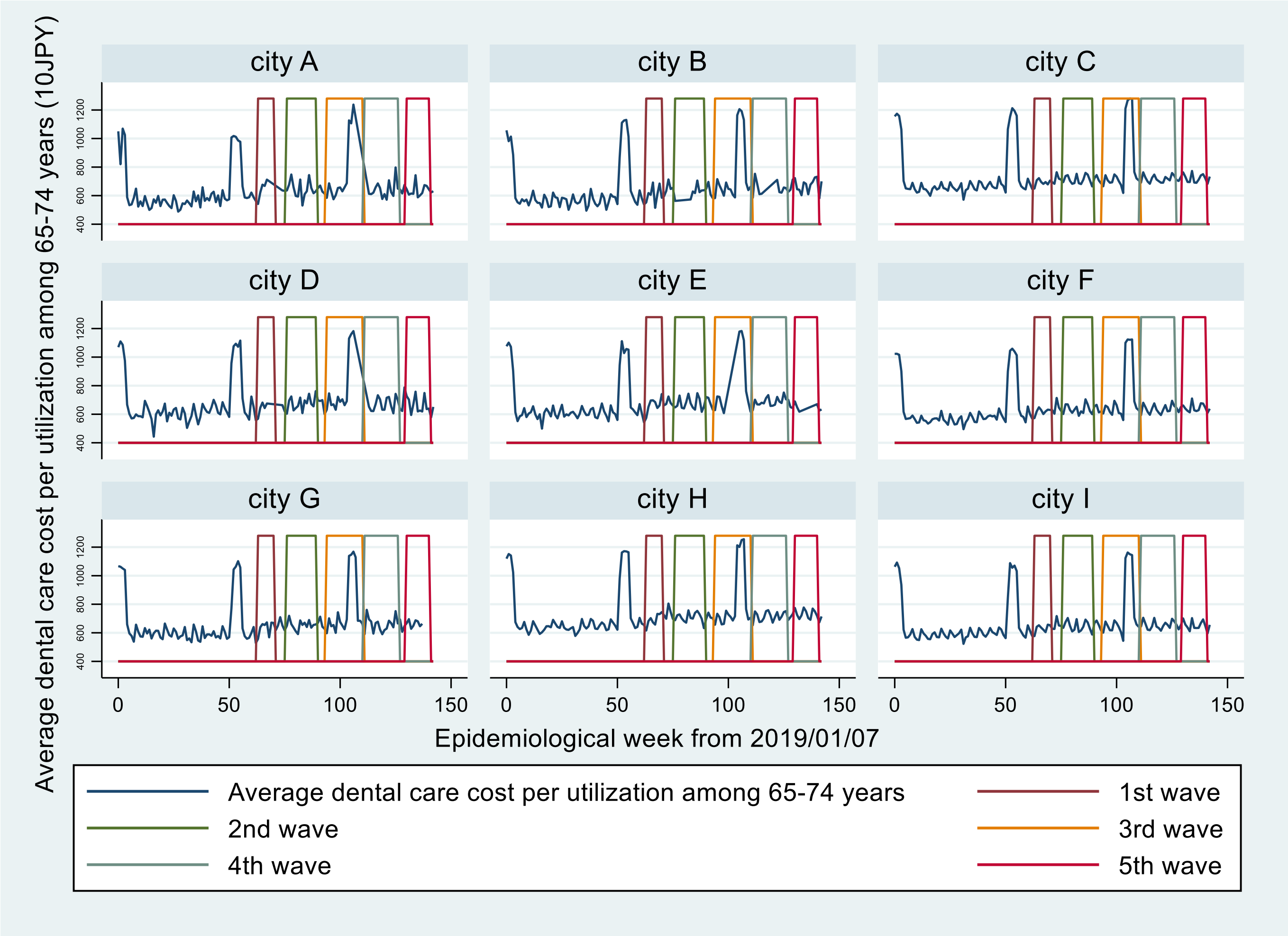

eFigure 15. The average dental care cost per attendance among the 65–74-year age group by municipality

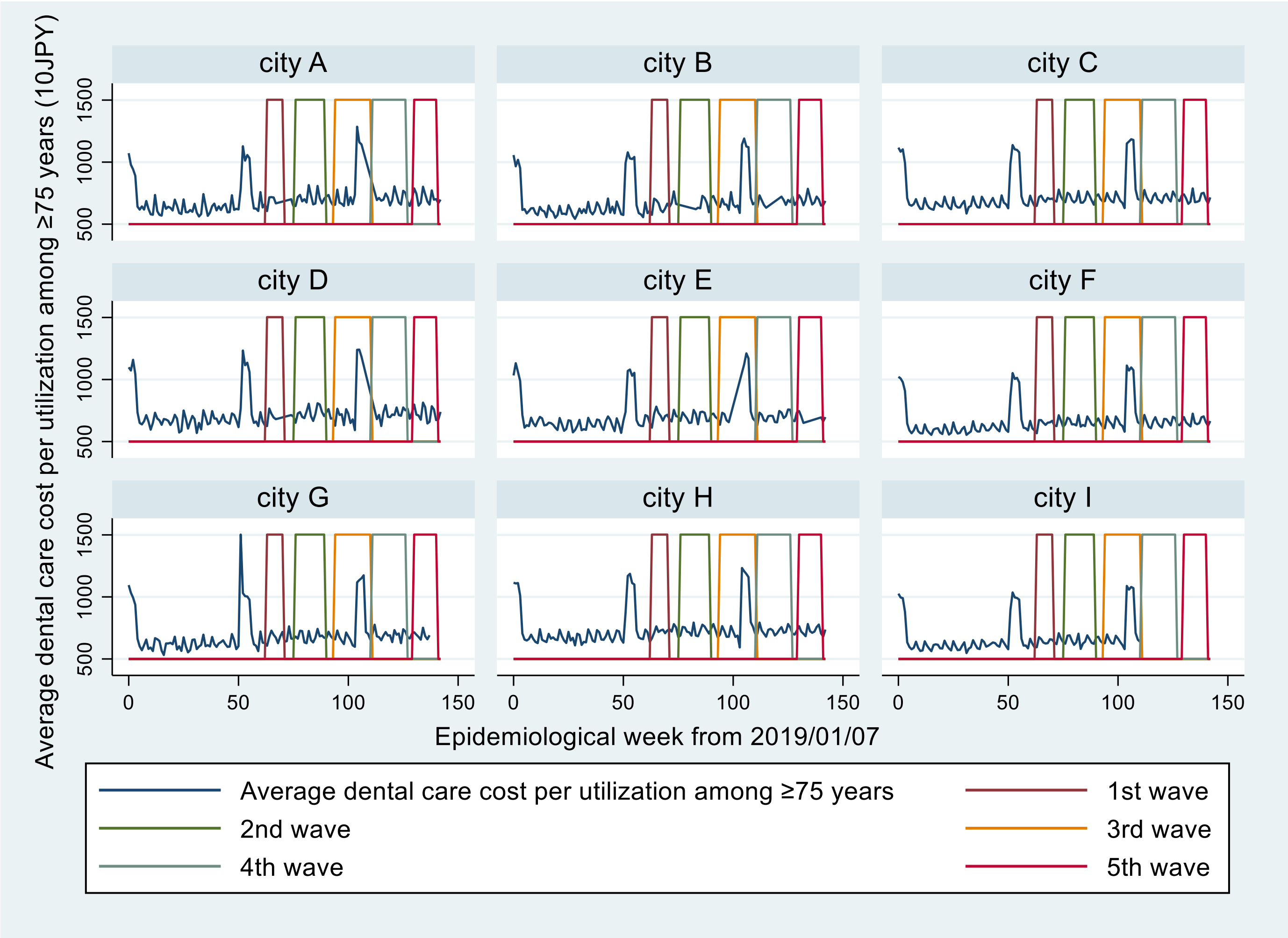

eFigure 16. The average dental care cost per attendance among the ≥75-year age group by municipality

eTable 2. The association between the COVID-19 pandemic and change in dental care utilization rate per week among nine municipalities with imputed datasets

Age groups	0–19 years[a]	20–64 years	65–74 years		≥75 years	
Types of dental care utilization	Total	Total	Outpatient dental care	Visiting dental care	Outpatient dental care	Visiting dental care
Pandemic waves	RR (95% CI)[b]	RR (95% CI)[b]	RR (95% CI)[b]	RR (95% CI)[b]	RR (95% CI)[b]	RR (95% CI)[b]
Non-pandemic wave period	1 (Ref.)	1 (Ref.)	1 (Ref.)	1 (Ref.)	1 (Ref.)	1 (Ref.)
1st wave	0.83 (0.80–0.86)	0.80 (0.78–0.83)	0.78 (0.75–0.81)	0.84 (0.80–0.89)	0.78 (0.75–0.82)	0.79 (0.76–0.83)
2nd wave	0.87 (0.82–0.92)	0.96 (0.92–0.99)	0.93 (0.89–0.98)	0.97 (0.92–1.02)	0.92 (0.88–0.96)	0.88 (0.84–0.92)
3rd wave	1.17 (1.11–1.22)	1.06 (1.02–1.09)	1.04 (1.00–1.08)	1.02 (0.97–1.07)	1.05 (1.01–1.10)	1.03 (0.97–1.08)
4th wave	1.12 (1.05–1.18)	1.07 (1.02–1.12)	1.06 (1.00–1.12)	1.05 (0.99–1.11)	1.08 (1.02–1.14)	1.11 (1.04–1.18)
5th wave	1.09 (1.02–1.16)	1.04 (0.98–1.09)	1.03 (0.97–1.11)	1.11 (1.04–1.18)	1.06 (0.99–1.13)	1.10 (1.03–1.17)

CI, confidence interval; COVID-19, coronavirus disease 2019; Ref., reference; RR, rate ratio.

[a] For age group of 0–19 years, data were obtained from eight municipalities.

[b] Estimates were adjusted for linear temporal trend, holidays in week, seasonal trends based on trigonometric functions.

Periods of pandemic waves were defined as follows: 1st wave (2020/03/23–2020/05/17), 2nd wave (2020/06/22–2020/09/27), 3rd wave (2020/10/26–2021/02/21), 4th wave (2021/02/22–2021/06/07), and 5th wave (2021/07/05–2021/09/13).

eTable 3. The association between the COVID-19 pandemic and change in dental care utilization rate per week among nine municipalities with complete records

Age groups	0–19 years[a]	20–64 years	65–74 years		≥75 years	
Types of dental care utilization	Total	Total	Outpatient dental care	Visiting dental care	Outpatient dental care	Visiting dental care
Pandemic waves	RR (95% CI)[b]	RR (95% CI)[b]	RR (95% CI)[b]	RR (95% CI)[b]	RR (95% CI)[b]	RR (95% CI)[b]
Non-pandemic wave period	1 (Ref.)	1 (Ref.)	1 (Ref.)	1 (Ref.)	1 (Ref.)	1 (Ref.)
1st wave	0.82 (0.79–0.86)	0.80 (0.77–0.82)	0.78 (0.75–0.81)	0.84 (0.80–0.89)	0.78 (0.75–0.81)	0.79 (0.75–0.83)
2nd wave	0.87 (0.82–0.92)	0.95 (0.92–0.99)	0.93 (0.89–0.98)	0.96 (0.92–1.02)	0.92 (0.88–0.96)	0.87 (0.83–0.92)
3rd wave	1.17 (1.11–1.22)	1.05 (1.02–1.09)	1.04 (1.00–1.08)	1.02 (0.98–1.07)	1.05 (1.01–1.10)	1.02 (0.97–1.08)
4th wave	1.11 (1.05–1.18)	1.07 (1.02–1.12)	1.06 (1.00–1.13)	1.05 (0.99–1.11)	1.09 (1.02–1.15)	1.10 (1.04–1.18)
5th wave	1.09 (1.02–1.16)	1.04 (0.98–1.09)	1.03 (0.96–1.11)	1.11 (1.04–1.18)	1.06 (0.97–1.15)	1.10 (1.02–118)

CI, confidence interval; COVID-19, coronavirus disease 2019; Ref., reference; RR, rate ratio.

[a] For age group of 0–19 years, data were obtained from eight municipalities.

[b] Estimates were adjusted for linear temporal trend, holidays in week, seasonal trends based on trigonometric functions.

Periods of pandemic waves were defined as follows: 1st wave (2020/03/23–2020/05/17), 2nd wave (2020/06/22–2020/09/27), 3rd wave (2020/10/26–2021/02/21), 4th wave (2021/02/22–2021/06/07), and 5th wave (2021/07/05–2021/09/13).

eTable 4. The association between the COVID-19 pandemic and change in average dental care cost per attendance among nine municipalities with imputed datasets

Outcome: Average dental care cost per attendance (JPY)				
Age groups	0–19 years[a]	20–64 years	65–74 years	≥75 years
Pandemic waves	Coef. (95% CI)[b]	Coef. (95% CI)[b]	Coef. (95% CI)[b]	Coef. (95% CI)[b]
Non-pandemic wave period	0 (Ref.)	0 (Ref.)	0 (Ref.)	0 (Ref.)
1st wave	477 (187–767)	695 (471–918)	653 (456–850)	636 (462–809)
2nd wave	-16 (-174 to 141)	135 (-1 to 271)	197 (58–336)	162 (41–284)
3rd wave	-283 (-571 to 5)	-205 (-377 to -32)	-144 (-356 to 68)	-163 (-361 to 35)
4th wave	320 (30–609)	261 (46–475)	261 (52–471)	266 (68–465)
5th wave	11 (-195 to 217)	-22 (-248 to 203)	-22 (-212 to 168)	-11 (-219 to 197)
**Outcome: Log-transformed average dental care cost per attendance (JPY)**				
Age groups	0–19 years[a]	20–64 years	65–74 years	≥75 years
Pandemic waves	% change (95% CI)[b]	% change (95% CI)[b]	% change (95% CI)[b]	% change (95% CI)[b]
Non-pandemic wave period	0 (Ref.)	0 (Ref.)	0 (Ref.)	0 (Ref.)
1st wave	5.2 (-0.1 to 10.6)	8.6 (4.8–12.3)	8.4 (5.0–11.7)	8.1 (5.4–10.8)
2nd wave	0.2 (-2.2 to 2.8)	2.8 (0.7–4.9)	3.9 (1.7–6.2)	3.0 (1.2–4.8)
3rd wave	-3.3 (-7.4 to 0.8)	-2.8 (-5.2 to -0.3)	-2.3 (-5.4 to 0.8)	-2.6 (-5.0 to -0.1)
4th wave	4.5 (-0.5 to 9.6)	3.6 (0.0–7.1)	3.6 (0.2–7.0)	3.6 (0.5–6.6)
5th wave	0.8 (-2.7 to 4.2)	0.5 (-3.0 to 4.0)	0.5 (-2.6 to 3.6)	0.4 (-2.8 to 3.6)

CI, confidence interval; Coef., coefficient; COVID-19, coronavirus disease 2019; JPY, Japanese yen; Ref., reference.

[a] For age group of 0–19 years, data were obtained from eight municipalities.

[b] Estimates were adjusted for linear temporal trend, holidays in week, seasonal trends based on trigonometric functions. Each estimate were presented in JPY.

Periods of pandemic waves were defined as follows: 1st wave (2020/03/23–2020/05/17), 2nd wave (2020/06/22–2020/09/27), 3rd wave (2020/10/26–2021/02/21), 4th wave (2021/02/22–2021/06/07), and 5th wave (2021/07/05–2021/09/13).

eTable 5. The association between the COVID-19 pandemic and change in average dental care cost per attendance among nine municipalities with complete records

Outcome: Average dental care cost per attendance (JPY)				
Age groups	0–19 years[a]	20–64 years	65–74 years	≥75 years
Pandemic waves	Coef. (95% CI)[b]	Coef. (95% CI)[b]	Coef. (95% CI)[b]	Coef. (95% CI)[b]
Non-pandemic wave period	0 (Ref.)	0 (Ref.)	0 (Ref.)	0 (Ref.)
1st wave	499 (307–691)	718 (597–838)	672 (577–768)	655 (602–707)
2nd wave	-29 (-127 to 70)	128 (42–213)	192 (98–285)	150 (78–222)
3rd wave	-275 (-541 to -10)	-198 (-310 to -85)	-140 (-304 to 24)	-164 (-332 to 3)
4th wave	332 (97–566)	269 (133–404)	269 (125–413)	263 (141–385)
5th wave	-4 (-110 to 101)	-37 (-144 to 61)	-38 (-92 to 15)	-32 (-94 to 29)
Outcome: Log-transformed average dental care cost per attendance (JPY)				
Age groups	0–19 years[a]	20–64 years	65–74 years	≥75 years
Pandemic waves	% change (95% CI)[b]	% change (95% CI)[b]	% change (95% CI)[b]	% change (95% CI)[b]
Non-pandemic wave period	0 (Ref.)	0 (Ref.)	0 (Ref.)	0 (Ref.)
1st wave	5.7 (2.8–8.6)	8.9 (7.1–10.7)	8.7 (7.1–10.3)	8.3 (7.6–9.1)
2nd wave	0.0 (-1.4 to 1.6)	2.6 (1.4–3.9)	3.8 (2.3–5.3)	2.8 (1.7–3.8)
3rd wave	-3.6 (-7.1 to 0.0)	-3.1 (-4.4 to -1.7)	-2.6 (-4.9 to -0.3)	-2.8 (-4.7 to -1.0)
4th wave	4.5 (0.9–8.1)	3.5 (1.7–5.3)	3.6 (1.7–5.5)	3.5 (1.9–5.0)
5th wave	0.5 (-1.0 to 2.1)	0.4 (-0.9 to 1.8)	0.4 (-0.5 to 1.4)	0.2 (-1.7 to 1.1)

CI, confidence interval; Coef., coefficient; COVID-19, coronavirus disease 2019; JPY, Japanese yen; Ref., reference.

[a] For age group of 0–19 year, data were obtained from eight municipalities.

[b] Estimates were adjusted for linear temporal trend, holidays in week, seasonal trends based on trigonometric functions. Each estimate were presented in JPY. Periods of pandemic waves were defined as follows: 1st wave (2020/03/23–2020/05/17), 2nd wave (2020/06/22–2020/09/27), 3rd wave (2020/10/26–2021/02/21), 4th wave (2021/02/22–2021/06/07), and 5th wave (2021/07/05–2021/09/13).
